# One-stage parametric meta-analysis of time-to-event outcomes

**DOI:** 10.1002/sim.4086

**Published:** 2010-10-20

**Authors:** F Siannis, J K Barrett, V T Farewell, J F Tierney

**Affiliations:** a Department of Mathematics, University of AthensGreece; b MRC Biostatistics UnitCambridge, U.K.; c MRC Clinical Trials UnitLondon, U.K.

**Keywords:** individual patient data, meta-analysis, extended log-gamma model, proportional hazards, time-to-event outcomes

## Abstract

Methodology for the meta-analysis of individual patient data with survival end-points is proposed. Motivated by questions about the reliance on hazard ratios as summary measures of treatment effects, a parametric approach is considered and percentile ratios are introduced as an alternative to hazard ratios. The generalized log-gamma model, which includes many common time-to-event distributions as special cases, is discussed in detail. Likelihood inference for percentile ratios is outlined. The proposed methodology is used for a meta-analysis of glioma data that was one of the studies which motivated this work. A simulation study exploring the validity of the proposed methodology is available electronically. Copyright © 2010 John Wiley & Sons, Ltd.

## 1. Introduction

Meta-analysis of data from multiple studies of the same research question has achieved a very high profile in medical research over the recent years. Currently, particular attention is being given to the potential value of individual patient data (IPD) 1 and the need to handle the challenges of meta-analysis of time-to-event outcomes.

Aggregate or summary data, such as hazard ratios and confidence intervals, can be used for time-to-event outcomes and they are commonly available in published papers. Methods for synthesizing evidence of this type (see discussion in 2), are borrowed from the methods used for summary statistics for simpler outcomes. However, in addition to the need for caution when extracting summary statistics of interest from papers or reports where they may not be clearly presented 3, this leaves little opportunity to examine the many characteristics of time-to-event data that may influence the results of standard analyses. To deal with this restriction, Fiocco *et al*. 4 have reconstructed data from the literature and provided a way to examine time-varying hazard models, an important generalization of what is normally possible with summary data. Other aspects of time-to-event data such as covariate adjustment may however be less easily handled with this approach. Thus, while IPD is considered the gold standard in meta-analysis in general 1, as all the relevant data are utilized, and approximations needed for aggregate data meta-analyses are avoided, their use is even more to be preferred with time-to-event outcomes for which a variety of distributional aspects may be of interest.

Simmonds *et al*. review methods used in the meta-analysis of IPD from randomized trials 5 and Tudur-Smith *et al*. explore the heterogeneity of IPD meta-analysis using hierarchical Cox regression models 6. The logarithm of the hazard ratio (logHR) is the most prevalent summary measure used in the meta-analysis of time-to-event endpoints. Although some argue that it is always justified to consider the logHR with time-to-event data, this approach is most natural in the presence of a proportional hazards (PH) structure 7. However, in a meta-analysis, the PH assumption can be particularly restrictive, since it is imposed on multiple studies. Fiocco *et al*. have provided a means to consider time-varying hazard ratios but there remains scope to consider the potential value of other approaches.

Here, the use of parametric models for meta-analysis of time-to-event IPD is explored as an alternative to the widely used Cox's PH model. Greater flexibility in the representation of treatment effects may be one advantage. Depending on the choice of model, various data structures can be naturally incorporated, with the accelerated failure time (AFT) structure being the most obvious alternative to the PH one. In principle, the combination of quite different data structures is possible since likelihoods of different forms from multiple studies can be combined to provide a basis for inference^8^. In addition, the use of a parametric model allows straightforward incorporation of covariates.

If we do not want to only consider models with a PH structure, the logHR cannot be adopted as the target of inference. As an alternative, we propose the use of a convenient ratio of percentiles, typically related to two treatment groups being compared, and which has the added advantage that it is defined for all distributions. An obvious choice is the median ratio. More generally, the percentile ratio (PR) can be regarded as a continuous function of the percentile. In this case, we can consider the *k*-PR, the ratio of the survival distributions at the *k*th percentile, as one of a possible set of measures of the treatment effect.

For illustration, we focus on AFT distributions defined in the extended log-gamma distribution, initially presented by Prentice in 9. For this family of distributions, the PR does not vary with the percentile chosen and is equal to the acceleration factor for the AFT models and can also be shown to be equal to the exponentiation of the treatment effect. These are also considered in combination with a PH model with log-logistic baseline, a model which does not have a constant PR.

Other families of distributions could be considered (log F 9, log Burr 10), but our aim is simply to allow variation in the representation of PRs and a wide scope in the choice of parametric form. In particular, this allows distributional variation across studies which goes beyond that represented by random effects, or frailty, time-to-event models. These may be suitable for some multi-center trials or meta-analyses but typically only allow a random shift in one parameter across centers or studies. Note that while non-parametric estimation of percentiles is also possible, the generality of the parametric approach maintains considerable flexibility in distributional shapes while also enabling the incorporation of covariates into the meta-analysis in a natural manner.

We begin in Section 2 by introducing a motivating example of the meta-analysis of glioma studies. In Section 3, we consider the PR as a measure of treatment efficacy. Maximum likelihood inference is also considered. In Section 4 a discussion of AFT models as well as the details of the extended log-gamma model are presented, while Section 5 gives details of how AFT models can be combined with log-logistic PH models in a meta-analysis framework. A discussion about study heterogeneity is presented in Section 6 followed by the analysis of glioma data in Section 7. The paper concludes with a discussion in Section 8.

## 2. Motivation: glioma example

We consider, as an example, an IPD meta-analysis of 12 randomized controlled trials investigating the use of chemotherapy in patients with high-grade glioma 11. Patients in the treatment groups were treated with surgery, radiotherapy and chemotherapy, while patients in the control groups were treated with surgery and radiotherapy. In the original report hazard ratios were estimated for each trial using log-rank analyses, and pooled hazard ratios were calculated for various outcomes using the fixed effects model. The overall hazard ratio for time to death was found to be 1.18 (95 per cent CI 1.09–1.28) comparing the treatment group with the control group. This was one of the meta-analyses undertaken at the MRC(UK) Clinical Trials Unit which prompted the methodological investigation reported here.

**Figure 1:**
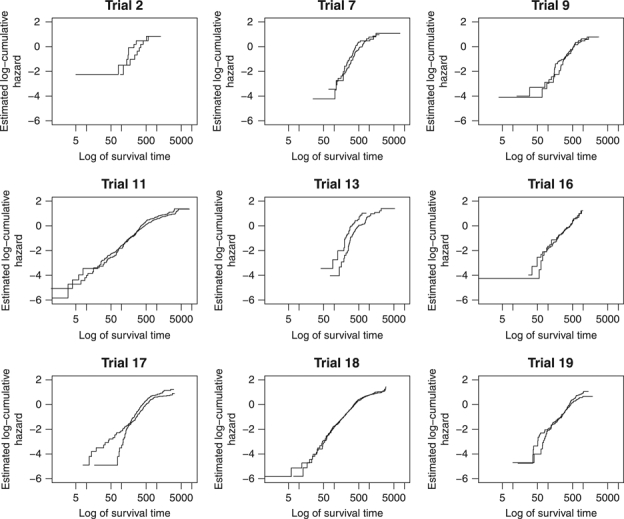
Assessing violations of Cox's PH assumption in the trials from the glioma data set.

The models we propose are alternatives to PH models. It is therefore interesting to examine the extent to which the PH assumption is violated in this data set. In Figure 1 for each trial the complementary log–log of the estimated survivor function has been plotted against the log of the survival times for the control and treatment groups, separately. Here the Kaplan–Meier estimate of the survivor function has been used. Under Cox's PH model the distance between the treatment and control curves in these graphs should be the same at all times. We can therefore assess by eye whether or not Cox's PH assumption is appropriate. From Figure 1 we conclude that the PH assumption is violated in some trials, the worst violation being in trial 17, while in others it appears appropriate, for example in trial 13.

In Section 7 we will reanalyze the glioma data using parametric models. We will initially restrict our attention to AFT models. The advantage of this approach lies in its simplicity, because for AFT models the PR is constant across percentiles (see Section 4.1). We will make use of the extended log-gamma distribution, which will be described in detail in Section 4.2, because it incorporates several common parametric AFT models for survival data, including the Weibull and log-normal distributions. Within this family it is not necessary to make a choice about the appropriate distribution for each trial but choices of distributions outside this family are possible. In order to illustrate this subsequently, we will choose to use log-gamma models for trials 9, 11, 17 and 19 while using a PH model with log-logistic baseline for the remaining trials for which a PH assumption appears most appropriate.

## 3. Measuring treatment efficacy

### 3.1. The percentile ratio

The *k*-PR *q*_*k*_ can be defined as

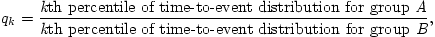
(1)
where *k* can take any value in [0, 1]. This quantity is thus relevant to any binary explanatory variable specifying group membership, such as a treatment identifier, and provides a relative measure for the treatment effect at each point on the survival probability axis. When discussing binary explanatory variables subsequently, we will assume that it is a treatment versus control comparison which is of interest. Note, however, that if a continuous explanatory variable is of interest, then *q*_*k*_ can be defined as the PR that reflects a unit change in the chosen variable. For *k* = 0.5, the quantity *q*_0.5_ represents the median ratio, possibly the percentile ratio of most general interest. Values >1 indicate, for example, that the median survival of the treatment group at this particular percentile is greater than the median survival of the control group, while values <1 indicate the opposite. In some circumstances, of course, another PR may be of more interest.

**Figure 2:**
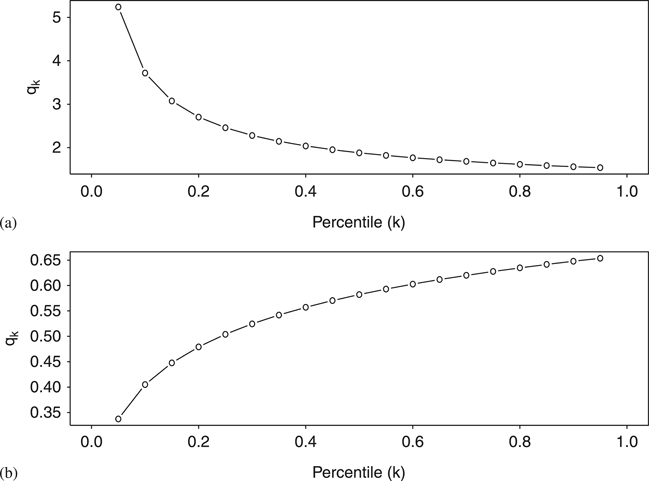
Plot of the PR for a proportional hazards distribution with log-logistic baseline, with positive (a) and negative (b) treatment effects.

In the most general setting, *q*_*k*_ changes as a function of *k*, since the PR for a specific value *k* does not capture the effect of treatment over the entire follow-up period of a trial. For that reason we might have to consider *q*_*k*_ over a range of values of *k*. For illustration, in Figure 2 the PR is plotted for a PH distribution with a log-logistic baseline, with (a) positive and (b) negative treatment effects. We consider percentiles only in the [0.05, 0.95] interval, since calculating *q*_*k*_ at the two limiting points, 0 and 1, is not informative. However, the limit for *k*→1 is calculable and can be taken to represent a final PR at the end of the study. At values of *k* close to zero, on the other hand, *q*_*k*_ is unstable, so conventionally we acknowledge that no treatment is better at *k* = 0, and hence *q*_0_ = 1. We thus adopt the notation *k*∈(0, 1) to imply that the extreme values 0 and 1 are not considered.

### 3.2. Likelihood inference

For now we focus on the inference concerning the PR *q*_*k*_ for a particular percentile level *k*. The situation when there is no natural or consensus choice of *k*, for inference purposes, is discussed later. Suppose we want to model the data from a study using a distribution *f*(*t*;*v*, β) for the time to an event *T*, where *v* is a parameter which characterizes the treatment effect, and β is a vector containing all other parameters relevant to the distribution. Irrespective of the choice of distribution *f*(), we can reparameterize it as *f*(*t*;*q*_*k*_, β) by expressing *v* as a function of *q*_*k*_, and possibly β, say *v* = *g*_*k*_(*q*_*k*_, β), conveniently written in this form to also highlight its dependence on the choice of *k*. However, *q*_*k*_ is a quantity with a clear interpretation and its scale does not depend on the choice of distribution *f*() or indeed any other features of the data being analyzed. Therefore, within a parametric meta-analysis, where different distributions are fit to data from different studies, *q*_*k*_ presents a measure of treatment effect in each of the separate analyses but remains directly comparable across studies. This means that, as a basis for meta-analysis, there exists a parameter common across distributions with an interpretation that can be easily communicated.

Consider now the case where we have *N* studies to be pooled for a meta-analysis and where we assume that *f*_*i*_(*t*;*b*_*i*_, *u*_*ij*_|*x*_*ij*_) is the chosen distributional form to model the data in study *i* (*i* = 1, …, *N*), where *j* (*j* = 1, …, *n*_*i*_) denotes the individuals in study *i*, *b*_*i*_ is a scale parameter and *u*_*ij*_ = µ_*i*_ + *v*_*i*_*x*_*ij*_ is the location parameter represented as a function of explanatory variables *x*_*ij*_, denoting treatment and other relevant patient-specific information. Still focussing on a particular percentile level *k*, we can express the distribution for study *i* as *f*_*i*_(*t*;µ_*i*_, *b*_*i*_, 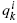
|*x*_*ij*_) using a reparameterization as discussed in the previous paragraph. Here 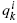 is the *k*-PR of study *i*. The most common assumption in a meta-analysis, of IPD or otherwise, is that the true value of the quantity of interest is the same across studies, while other parameter values can vary. Therefore, we fix 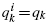 for all studies. Then, the likelihood function can be written as

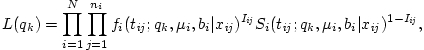
(2)
where *I*_*ij*_ is the usual indicator variable for events. Also, the usual assumption that censoring is non-informative, in each of the *N* studies, is made. Based on (2), standard maximum likelihood estimation (MLE) of the common parameter *q*_*k*_ is possible.

Previously in this section we have focussed on the inference concerning a particular value of the percentile level *k*. However, it may be more appropriate to consider a range of values of *k*. In this case we can carry out a separate analysis for each value of *k* and plot the results against *k*. Since the reparameterization procedure we used to derive the likelihood (2) may depend on *k*, for every choice of *k* there is a different likelihood. The likelihoods can only lead to identical inferences if the *q*_*k*_'s can be jointly modeled to be common across studies for every *k*, generally only true if *q*_*k*_ = *q*for all *k* or if the dependence of *q*_*k*_ on *k* is modeled to be the same across studies through an assumption of a common distributional shape. The second possibility will only be true under restrictive assumptions about the common features of the time-to-event distributions across trials. The first is less restrictive in regard to distributional shape and is, for example, satisfied if estimation is based on the log-gamma family of AFT distributions.

More generally, we suggest that 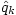, which is defined by maximization of (2) for a specific *k*, be regarded as a particular pooled summary of the 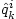 that would be calculated from the studies individually. Then 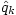, viewed as a continuous function of *k*∈(0, 1), represents these pooled summaries and will provide some indication of the variation of the treatment effect over *k*. The 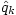 values, for separate values of *k*, will be correlated but there appears to be no compelling practical reason to consider formal simultaneous inference that would depend on this correlation since separate 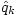 's are based on different model assumptions.

**Figure 3:**
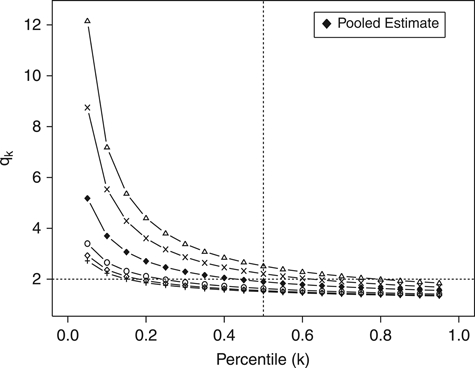
Plot of data generated from PH log-logistic distributions for five studies with different parameter values but a common median percentile ratio *q*_0.5_ = 2. The pooled estimate has also been plotted.

For illustration, Figure 3 presents a simple case where, for five different studies, we have generated data from PH log-logistic distributions, as in the example in Figure 2. There was no censoring and the data were generated based on different sets of parameters, with the common characteristic *q*_.5_ = 2. Clearly *q*_*k*_ for values of *k*≠0.5 is not the same across studies, especially for small *k*. Based on the meta-analysis framework introduced in this section, we obtain the pooled estimate 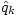, plotted for *k*∈[0.05, 0.95], which nicely falls in the center of individual study curves that give the estimated *q*_*k*_ values and thus summarizes them in a single curve. For *k* = 0.5 we get 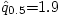, very close to the true value of 2.

## 4. Parametric models for meta-analysis

### 4.1. AFT models

A parametric AFT model for a random variable *Y* on (−inf*ty*, inf*ty*) corresponds to the use of a distribution with p.d.f.

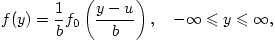

where *u*(−∞≤*u*≤∞) and *b*>0 are the location and scale parameters, respectively, *f*_0_(*z*) is a p.d.f on (−∞, ∞) and *Y* = log *T*, where *T* represents time. The distribution and survival functions for *Y* are *F*_0_[(*y* − *u*)/*b*] and *S*_0_[(*y* − *u*)/*b*] respectively, where

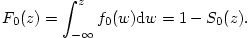

The survival function for *T* = exp(*Y*) can be expressed as

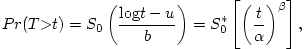

where α = exp(*u*), β = *b*^−1^ and 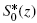 is the survival function defined by the relationship 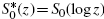. For treatment comparisons based on a treatment indicator *x*, we would let *u* = *u*(*x*) = µ + *vx*. For a more detailed discussion of log-location-scale models see 12. This model can also be expressed as a regression model

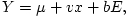
(3)
where *E* is a random variable with p.d.f. *f*_0_(*z*). This has been a very useful model for the parametric analysis of time-to-event data. Extreme value, normal and logistic distributions for *Y*, correspond to Weibull, log-normal and log-logistic distributions for *T*, which are three of the most popular distributions for this purpose.

For this model, if 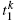 and 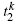 are *k*th percentiles for the time-to-event distribution in treatment and control groups, respectively, then

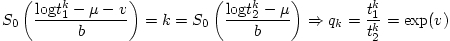
(4)
and thus *q*_*k*_ does not depend on *k*. This is a known and expected result for AFT models as the explanatory variables act multiplicatively on the time scale, and *q*_*k*_ is equal to the *acceleration factor* for all *k*. If every distribution *f*_*i*_(*t*;*b*_*i*_, *u*_*ij*_|*x*), as defined in Section 3.2, has this AFT structure, then the reparameterization as suggested in (4), is trivial and the assumption that PRs are constant across studies is simply an assumption that regression coefficients are the same across studies. The estimation of *q*_*k*_, which equals a common value *q* for all *k*, can then be based on a single likelihood defined by (2).

Another advantage of restricting attention to AFT models is that it is straightforward to include covariates, by adding extra terms to the right-hand side of equation (3). Within an IPD meta-analysis, patient-level covariates can be added in one of two ways. The covariate effects can be constrained to be the same in all studies or they can be allowed to vary across studies. In the former case the assumption of equal covariate effects can be tested by using a likelihood ratio test to compare the two models.

### 4.2. The extended log-gamma model

A useful class of AFT models is represented by the extended log-gamma model. This is a regression model of the form (3), where the error p.d.f. is written as

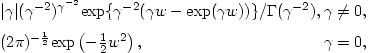

which is a representation of Γ(κ)^−1^exp(κ*u* − exp(*u*)), the log-gamma distribution, following some model manipulation and with *w* = (*Y* − µ − *vx*)/*b*. The survival function *S* is given by

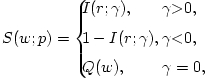

where *r* = γ*w* − 2ln(γ),

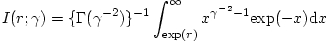

is the incomplete gamma ratio and

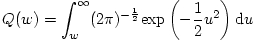

is the incomplete normal integral. This distribution was initially discussed in 9, where an extension to the log-gamma model was considered by allowing γ<0, with the p.d.f. at −γ being a reflection about the origin of that at γ. Further discussion about this model can be found in 13, together with illustrative applications and additional references. A recent tutorial paper 14 on the use of the generalized gamma distribution for survival analysis has also appeared.

This three-parameter family of distributions, where the parameter γ, together with *b*, specifies the form of the error density function, includes the special cases for *T* = e^*Y*^ of Weibull (γ = 1), exponential (γ = *b* = 1), log-normal (γ = 0), gamma (γ = *b*), generalized gamma (γ>0) and reciprocal Weibull (γ = − 1) distributions. More generally, through estimation of γ, we minimize the need for assumptions about error distributions in various studies being considered. Of course, there is a need to estimate the *N* γ parameters but this should not be a problem if each study is of moderate size as evidenced by the examples in 13.

In an electronic appendix‡ we investigate the empirical behavior of meta-analyses based on PRs for the special case of the log-gamma family of distributions and demonstrate that it performs as expected. We compare the extended log-gamma model to models which assume all Weibull, log-normal or log-logistic distributions, and find that the PR standard errors are smaller for the extended log-gamma model because it provides a better fit to the data through allowing the underlying time-to-event distributions to vary across studies.

Since Weibull distributions are linked to PH models of widely varying shapes and retain the AFT assumption of common PRs, the log-gamma family was convenient for our simulation study and, more generally, may represent a useful approach to the meta-analysis of multiple trials where not all will necessarily be consistent with a PH representation of a treatment effect. However, to illustrate that this is not a necessary restriction we consider an alternative PH family in the following section.

## 5. Incorporating log-logistic PH models

In this section, we consider the more complicated situation when AFT models are appropriate for some studies whereas PH models which are not also in the AFT class are appropriate for others. For this purpose, we can, as was done to produce Figure 2, consider a PH model *h*(*t*;θ|*x*) = e^θ*x*^*h*_0_(*t*) with log-logistic baseline function, where

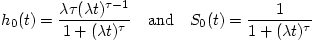

are the baseline hazard and survival functions, respectively. It can then be easily shown that

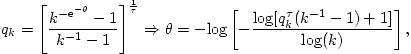
(5)
which makes explicit that the relationship between θ, the logHR, and the PR is a function of *k*.

If (5) is used to reparameterize this PH model, then we can use both AFT and PH models in the likelihood (2) to make inferences concerning *q*_*k*_. Because the inclusion of PH models means that *q*_*k*_ will depend on *k*, we could, as suggested earlier, plot 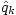 against *k* or focus on a particular value of *k* of interest.

## 6. Heterogeneity

Exploring heterogeneity is always advisable in meta-analysis. It is very important to feel confident that summary inferences drawn from multiple studies are informative. In RCTs where only aggregate data are available it is natural to obtain a forest plot and observe whether the estimates of the treatment effects across trials are similar. The parametric scenario, presented in this paper, provides a useful structure in order to test whether PRs are the same across studies. Along with the production of a forest plot, we can easily test the null hypothesis 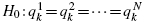 against the alternative of arbitrary differences based on a likelihood ratio test with *N* − 1 degrees of freedom. This test examines heterogeneity at the *k*-percentile, giving some information about the homogeneity of the studies. As for the summary measures themselves, when PRs vary with *k* and multiple tests can be performed, formal simultaneous inference concerning heterogeneity is unlikely to be of central interest.

## 7. Analysis of glioma example

In this section we return to the example described in Section 2. We were unable to obtain permission to use the data from 3 of the 12 trials in the original meta-analysis. We will therefore use data from nine trials in our analysis.

Model fitting used the R software for statistical analysis (http://www.r-project.org), in which code was written to generate and analyze the data (available in the electronic appendix). Of readily available alternative software, STATA may be an attractive alternative since its parametric survival regression package includes the extended log-gamma distribution as one of the default distributions. Furthermore, it allows all ancillary parameters to be estimated separately in each study while restricting regression coefficients to be the same across studies. However, it does fit the same distribution for all studies and would also restrict regression coefficients other than that associated with treatment to be the same across studies. These are possible drawbacks.

**Figure 4:**
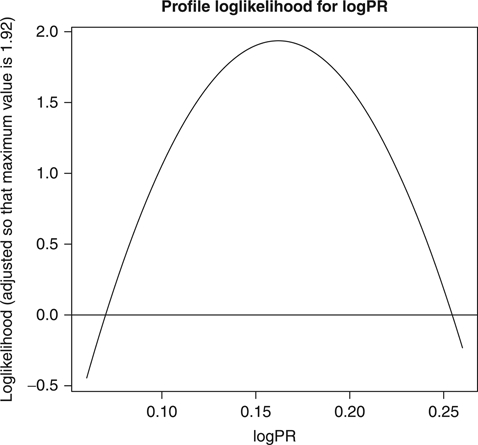
Profile log-likelihood plot for the logPR for the glioma data analysis using the extended log-gamma model.

We analyze the data initially using AFT models. We use the extended log-gamma distribution, taking advantage of its flexible nature by allowing the shape parameter to vary between trials. The location and scale parameters of the log-gamma distribution are also allowed to vary between trials, while the logarithm of the percentile ratio (logPR) is assumed to be the same in all trials. We obtain parameter estimates by maximizing the likelihood (2) over all parameters simultaneously, using optim in R and their asymptotic variance–covariance matrix was estimated by the inverse of the observed information matrix. The pooled logPR estimate for the glioma data is 0.163 (95 per cent CI 0.071, 0.255), which corresponds to a PR of 1.176. Recall that for AFT models the PR is constant across percentiles. The PR can therefore be explained as the ratio of the survival time for a patient on the experimental treatment to the survival time for a patient on the control treatment for any percentile. A profile likelihood plot for the logPR is shown in Figure 4 to demonstrate that the likelihood is symmetric about the estimated logPR. In Figure 4 a constant has been added to the loglikelihood so that the maximized value is 1.92; confidence intervals are therefore given by the intersection of the curve with the *x*-axis.

**Figure 5:**
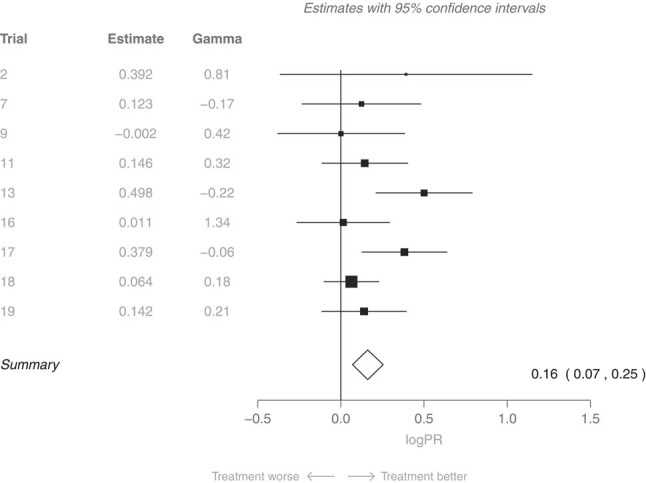
Forest plot for the ELG meta-analysis of the glioma data. The table gives the estimated logPR for each trial, along with the estimated value of the shape parameter for each trial.

A forest plot for the glioma data based on the extended log-gamma model is shown in Figure 5, along with estimates of the logPRs and shape parameters from each trial. In this plot the individual trial estimates derive from an extended log-gamma analysis of each study individually. The test for heterogeneity described in Section 6 gives a likelihood ratio statistic of 11.4 for the glioma data, which corresponds to a *p*-value of 0.18 when compared with a chi-squared distribution with eight degrees of freedom.

We demonstrate the addition of patient-level covariates here by adjusting the model for sex. As described in Section 4.1, this can either be done by constraining the covariate effect to be the same in all trials, or by allowing it to vary between trials. The first method, with identical covariate effects in all trials, gives an estimated logPR of 0.20 (95 per cent CI 0.12, 0.28), which is slightly higher than the unadjusted estimate of 0.16. Allowing covariate effects to vary between trials, the estimated logPR is 0.19 (95 per cent CI 0.11, 0.27). The two methods therefore give similar results in this case, although a likelihood ratio test comparing the two gives a chi-squared statistic of 18.49 on eight degrees of freedom, which corresponds to a *p*-value of 0.018. This suggests that use of the more complicated model is justified here. Other covariates which may have been interesting to include in this example are histology and the extent of resection, but these have been omitted due to the presence of missing data.

In the second analysis of the same data set we use a combination of PH models with log-logistic baselines and AFT models. In Figure 1 we presented some diagnostic plots which can be used to assess whether or not a PH assumption is appropriate for the data from each trial. On the basis of these plots we model the data from trials 9, 11, 17 and 19 using AFT distributions and the data from the remaining trials using log-logistic PH distributions. As before we will take the PR to be constant across trials. However, because the PR may now vary across percentiles, we must impose this assumption separately for each percentile. This means that we are effectively fitting a different model at each percentile. We maximize the likelihood (2) for each percentile using the extended log-gamma distribution for data from the first set of trials, and the log-logistic PH distribution, described in Section 5, for data from the remaining trials. For the PH distributions, equation (5) is used to convert the logHR into the logPR.

**Figure 6:**
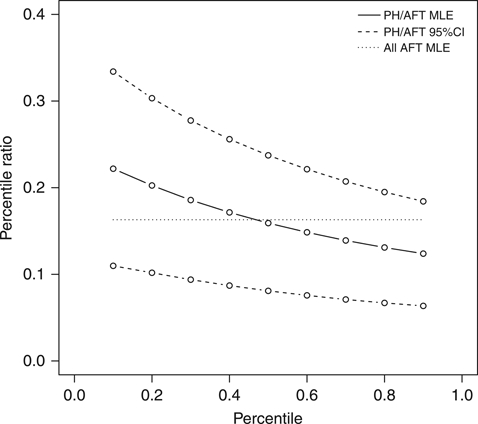
Results of the PH/AFT analysis—estimated logPR's are plotted as a solid line and 95 per cent confidence intervals around these estimates are plotted as dotted lines. The circles indicate the percentiles at which logPR's were estimated. The estimated logPR from the analysis which assumes AFT models for all trials is plotted as a dotted line.

The results from fitting the PH/AFT model are presented in the graph in Figure 6. Estimates for the pooled logPR are plotted as a solid line, with 95 per cent confidence intervals as dashed lines. The logPR was estimated for percentiles at intervals of 0.1, indicated by circles on the curves in the graph. The estimated logPR decreases as the percentile increases, which is a consequence of using the log-logistic hazard function as a baseline in the PH models. Note that the curve is fairly flat, which means that an analysis assuming AFT models for all trials may be justified in this case. Also plotted as a dotted line in Figure 6 is the estimated logPR from the first analysis. This result lies in the middle of the PH/AFT results, coinciding with the PH/AFT curve around the median percentile ratio.

Our results are in keeping with the finding of the original meta-analysis of this data set, which however used data from all 12 trials. The original result was a pooled hazard ratio of 1.18 comparing radiotherapy plus chemotherapy to radiotherapy alone. This corresponds to a logHR of 0.16, which is very close to our pooled AFT logPR estimate of 0.16. The PR estimate has the advantage that its meaning is easier to interpret for practitioners, being the estimated increase in survival time for those in the experimental group compared with those in the control group.

## 8. Discussion

In this paper, our aim was to discuss the potential value of percentile ratios in the IPD meta-analysis of time-to-event outcomes. Because percentile ratios can be defined for the comparison of any two survival curves, they provide a reasonable basis on which to compare and combine treatment effects across studies. For this reason, we feel they are worth investigation as a basis for IPD meta-analysis of time-to-event outcomes. Ultimately their advantages and disadvantages with respect to analyses based on hazard ratios can only emerge as they are used in a variety of settings.

When each study is modeled using the correct distribution and the PR of interest, *q*_*k*_, is common across studies, then MLE will provide unbiased estimates for this PR. Even if percentile ratios from different studies, the 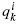
's, vary then 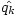 will still be a potentially useful pooled summary of these values. Further work should explore the consequences of model misspecification more generally however.

Different time-to-event models may be adopted for different studies, but the extended log-gamma model represents a broad class of distributions which may be particularly useful. An AFT family of distributions has the particular advantage that the PR is invariant across the percentile level. An analysis based on extended log-gamma models is therefore simple and relatively undemanding computationally, and provides a single estimate of treatment effect which is easily interpretable.

Alternatively, distributions may be considered for which the PR does depend on the percentile level. Then it may be that a single or a set of percentile levels is of interest. The choice is likely to be context specific. Here, we have focussed on the use of PH distributions with log-logistic baseline hazard functions in combination with AFT distributions. In practice, however, alternative distributions for the baseline hazard could be considered. This is a more flexible approach than assuming AFT distributions for all studies, but more work is required to investigate how the possibly subjective choice of distribution for each study might affect the results. Here, we have used graphical methods to assess departure from the PH assumption, but with a larger data set more sophisticated methods such as those proposed by Boutitie *et al*. 15 might be used.

Subsequently, our aim is to investigate random effects models in this context in which the PRs from different studies are assumed to come from a known distribution. A possible hierarchical regression model, for this purpose, could introduce random effects *v*_*ik*_ as follows:

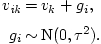
(6)
*v*_*k*_ = log (*q*_*k*_) is considered to be the average log-PR and *g*_*i*_ is the deviation from the log-PR in study *i*. Thus the *q*_*k*_'s are no longer assumed to be the same across studies but instead are assumed to come from a Normal distribution with mean *v*_*k*_ and variance τ^2^. The WinBugs software can be used to fit random effects models with known distributions for each study, and these need not be AFT. However, the extended log-gamma model is not available by default in WinBugs and so separate development in this package or otherwise will be required.

## Footnotes

‡ Supporting information may be found in the online version of this article.

## Appendix A

### A.1. Simulation studies

In this section, we further explore and illustrate our proposed methodology through a simulation with the following structure. Data are generated for five independent studies, based on the extended log-gamma model, each of which have 200 patients, 100 in each treatment arm. Censoring is assumed to be random following an exponential distribution, going from 0 to 40 per cent per study. We assume that the constant PR is equal to 2 for every study, corresponding to the regression coefficient for the treatment effect being log (2) = 0.6931, while the remaining parameters of the error distribution are allowed to take various values, as seen in Table AI. Parameter values have been chosen to generate sets of studies with lesser or greater variation in the shape parameters (simulation A versus simulation B), and with shape and location parameters which are either constant or varying across studies. This procedure is repeated 500 times for every set of parameters and for every prespecified amount of censoring.

**Table AI:** Set of parameters for simulations with the extended log-gamma model.

	γ	μ	β
*Sim A1*	(0.3, 0.6, 0.9, 1.2, 1.5)	(7,7,7,7,7)	(1,1,1,1,1)
*Sim A2*	(0.3, 0.6, 0.9, 1.2, 1.5)	(4,9,7,3,8)	(1,1,1,1,1)
*Sim A3*	(0.3, 0.6, 0.9, 1.2, 1.5)	(4,9,7,3,8)	(1.5,0.6,1.2,0.8,1.1)
*Sim B1*	(−2, −1, 0.3, 1, 2)	(7,7,7,7,7)	(1,1,1,1,1)
*Sim B2*	(−2, −1, 0.3, 1, 2)	(4,9,7,3,8)	(1,1,1,1,1)
*Sim B3*	(−2, −1, 0.3, 1, 2)	(4,9,7,3,8)	(1.5,0.6,1.2,0.8,1.1)

Two sets of analyses are based on each of Weibull, log-normal and log-logistic distributions. The first is a regression analysis, stratified on study, while the second is an unstratified regression analysis based on the pooled data from all five studies, effectively assuming that they come from a single source. Of course, this latter approach would not generally be recommended but is included for comparison purposes. Since these are all AFT models, the MLE for the treatment effect should be unbiased, irrespective of any distributional assumption. In addition, an analysis is based on the extended log-gamma model, where the treatment effect is assumed to be common across studies, while all remaining parameters are allowed to vary across studies.

A subset of the results of the simulations are presented in Table AII and Table AIII, which focus on estimation of the regression coefficient associated with the treatment. Note that to avoid convergence problems in the three B simulations with no censoring when very large times could sometimes arise, all times in those simulations were truncated at 250 000. This will introduce some negative bias in the mean estimated treatment effect, most notably for the Weibull, but this should not have a substantial impact on comparative statements.

**Table AII:** Results of the A simulations, based on various levels of censoring. Estimates are given with standard deviations of estimates in curved brackets and mean estimated standard errors in square brackets.

	Stratified analysis			Single study analysis			
			
	Weib	LL	LN	Weib	LL	LN	ELG
*Sim A1* (0 per cent)	0.6969	0.6976	0.6977	0.6965	0.6974	0.6977	0.6971
	(0.0643)	(0.0749)	(0.0817)	(0.0654)	(0.0741)	(0.0817)	(0.0628)
	[0.0640]	[0.0759]	[0.0820]	[0.0652]	[0.0768]	[0.0843]	[0.0626]
*Sim A1* (20 per cent)	0.6903	0.6901	0.6895	0.6904	0.6903	0.6894	0.6916
	(0.0745)	(0.0848)	(0.0950)	(0.0748)	(0.0840)	(0.0956)	(0.0748)
	[0.0711]	[0.0827]	[0.0904]	[0.0724]	[0.0840]	[0.0931]	[0.0693]
*Sim A1* (40 per cent)	0.6884	0.6871	0.6876	0.6880	0.6873	0.6877	0.6894
	(0.0848)	(0.0929)	(0.1062)	(0.0850)	(0.0921)	(0.1079)	(0.0834)
	[0.0817]	[0.0928]	[0.1028]	[0.0832]	[0.0947]	[0.1065]	[0.0791]
*Sim A2* (0 per cent)	0.6947	0.6951	0.6960	0.6947	0.6970	0.6960	0.6948
	(0.0676)	(0.0794)	(0.0863)	(0.0897)	(0.0907)	(0.0863)	(0.0655)
	[0.0640]	[0.0757]	[0.0818]	[0.1440]	[0.1774]	[0.1670]	[0.0626]
*Sim A2* (20 per cent)	0.6991	0.6982	0.6966	0.6964	0.6991	0.6971	0.6987
	(0.0768)	(0.0859)	(0.0953)	(0.1127)	(0.1287)	(0.1213)	(0.0753)
	[0.0711]	[0.0827]	[0.0904]	[0.1612]	[0.1946]	[0.1864]	[0.0694]
*Sim A2* (40 per cent)	0.6981	0.6977	0.6991	0.6943	0.6988	0.6983	0.6965
	(0.0838)	(0.0945)	(0.1062)	(0.1492)	(0.1689)	(0.1644)	(0.0846)
	[0.0818]	[0.0929]	[0.1031]	[0.1867]	[0.2189]	[0.2148]	[0.0791]
*sim A3* (0%)	0.6981	0.7010	0.7013	0.6995	0.7022	0.7013	0.6961
	(0.0753)	(0.0757)	(0.0830)	(0.0687)	(0.0950)	(0.0830)	(0.0564)
	[0.0687]	[0.0785]	[0.0870]	[0.1422]	[0.1829]	[0.1688]	[0.0557]
*sim A3* (20 per cent)	0.6925	0.6948	0.6965	0.6984	0.7016	0.7012	0.6926
	(0.0763)	(0.0846)	(0.0966)	(0.1030)	(0.1327)	(0.1216)	(0.0638)
	[0.0746]	[0.0854]	[0.0954]	[0.1612]	[0.2011]	[0.1888]	[0.0617]
*sim A3* (40 per cent)	0.6946	0.6955	0.6954	0.6926	0.6942	0.6926	0.6927
	(0.0825)	(0.0888)	(0.1040)	(0.1384)	(0.1704)	(0.1590)	(0.0697)
	[0.0852]	[0.0964]	[0.1084]	[0.1904]	[0.2281]	[0.2190]	[0.0707]

**Table AIII:** Results of the B simulations, based on various levels of censoring. Estimates are given with standard deviations of estimates in curved brackets and mean estimated standard errors in square brackets.

	Stratified analysis			Single study analysis			
			
	Weib	LL	LN	Weib	LL	LN	ELG
*Sim B1** (0 per cent)	0.6343	0.7003	0.6910	0.6009	0.6996	0.6910	0.6972
	(0.1070)	(0.0816)	(0.0913)	(0.1512)	(0.0724)	(0.0913)	(0.0630)
	[0.0878]	[0.0870]	[0.0955]	[0.1170]	[0.0950]	[0.1125]	[0.0626]
*Sim B1* (20 per cent)	0.6739	0.6918	0.6892	0.6564	0.6922	0.6884	0.6926
	(0.0989)	(0.0896)	(0.1019)	(0.1396)	(0.0848)	(0.1059)	(0.0705)
	[0.0874]	[0.0912]	[0.1007]	[0.1134]	[0.1026]	[0.1200]	[0.0668]
*Sim B1* (40 per cent)	0.6966	0.6945	0.6948	0.6940	0.6935	0.6932	0.6925
	(0.0926)	(0.0911)	(0.1061)	(0.1133)	(0.0938)	(0.1169)	(0.0740)
	[0.0911]	[0.0977]	[0.1102]	[0.1136]	[0.1135]	[0.1332]	[0.0733]
*Sim B2* (0 per cent)	0.6405	0.6920	0.6877	0.6469	0.6943	0.6877	0.6960
	(0.1404)	(0.0793)	(0.0924)	(0.1161)	(0.1001)	(0.0924)	(0.0629)
	[0.0986]	[0.0876]	[0.0983]	[0.1591]	[0.1819]	[0.1758]	[0.0623]
*Sim B2* (20 per cent)	0.6979	0.6962	0.6987	0.6961	0.7020	0.7005	0.6963
	(0.1010)	(0.0895)	(0.1021)	(0.1374)	(0.1283)	(0.1259)	(0.0715)
	[0.0881]	[0.0916]	[0.1012]	[0.1750]	[0.1973]	[0.1936]	[0.0670]
*Sim B2* (40 per cent)	0.6913	0.6910	0.6921	0.6814	0.6893	0.6867	0.6963
	(0.0952)	(0.0963)	(0.1101)	(0.1613)	(0.1681)	(0.1658)	(0.0761)
	[0.0911]	[0.0975]	[0.1094]	[0.1984]	[0.2220]	[0.2204]	[0.0734]
*Sim B3** (0 per cent)	0.6215	0.6873	0.6785	0.6417	0.6858	0.6785	0.6902
	(0.1217)	(0.0887)	(0.1065)	(0.1249)	(0.1179)	(0.1065)	(0.0600)
	[0.1011]	[0.0934]	[0.1094]	[0.1553]	[0.1859]	[0.1762]	[0.0558]
*Sim B3* (20 per cent)	0.6884	0.6934	0.6922	0.6867	0.6888	0.6888	0.6944
	(0.1103)	(0.0918)	(0.1106)	(0.1256)	(0.1411)	(0.1334)	(0.0644)
	[0.0954]	[0.0983]	[0.1116]	[0.1648]	[0.1985]	[0.1913]	[0.0594]
*Sim B3* (40 per cent)	0.6987	0.6994	0.7027	0.7042	0.7043	0.7047	0.6983
	(0.0937)	(0.0946)	(0.1127)	(0.1371)	(0.1632)	(0.1577)	(0.0665)
	[0.0949]	[0.1025]	[0.1168]	[0.1890]	[0.2201]	[0.2154]	[0.0651]

* Event times have been truncated at 250 000.

As expected, all the point estimates have means very close to the true value 0.6931. In all simulations, the extended log-gamma analysis has standard errors comparable to or smaller than the others. The mean of the estimated standard errors from the log-gamma model is slightly smaller than the true value. This suggests that the asymptotic behavior of these maximum likelihood estimates is not quite being achieved with the sample sizes simulated but the practical import is minimal as differences are in the second significant digit.

For the *A* simulations, the estimated standard errors from the stratified regression analyses are also reasonably close to the actual standard errors and moderately comparable to those from the extended log-gamma model. This is due to the fact that the data structure, same or different means but the same scale parameter across studies and reasonably similar shape parameters, can be captured effectively by the stratified analysis. In contrast, the estimated standard errors from the single study analysis perform less well.

For the *B* simulations that have greater variation in the shape parameters, the performance of the misspecified models, where some trials are modeled incorrectly, is less acceptable. In all simulations, the standard errors from the misspecified models are larger than that from the extended log-gamma analysis although the estimated standard errors from the stratified analyses, although underestimates, are reasonably close to the true values.

Coverage probabilities are presented in Table AIV and the results reflect the behavior of the estimated standard errors in Table AII and Table AIII. For the extended log-gamma model the probabilities are close to the desired 0.95, although typically slightly less reflecting the underestimation of the standard error of the estimates. However, the coverage probabilities for the stratified and single study analyses can be as small as 0.792 and as high as 1.000 for different simulation scenarios. In general probabilities are closest to 0.95 when location and scale parameters are constant across studies.

**Table AIV:** Coverage probabilities.

	Stratified analysis			Single study analysis			
			
	Weib	LL	LN	Weib	LL	LN	ELG
*Sim A1* (0 per cent)	0.952	0.944	0.942	0.946	0.952	0.942	0.952
*Sim A1* (20 per cent)	0.934	0.942	0.934	0.940	0.944	0.938	0.928
*Sim A1* (40 per cent)	0.946	0.952	0.938	0.944	0.962	0.938	0.944
*Sim A2* (0 per cent)	0.930	0.938	0.934	0.998	0.998	0.998	0.930
*Sim A2* (20 per cent)	0.926	0.940	0.934	0.996	1.000	1.000	0.930
*Sim A2* (40 per cent)	0.952	0.940	0.944	0.990	0.994	0.994	0.932
*Sim A3* (0 per cent)	0.926	0.948	0.952	1.000	1.000	1.000	0.946
*Sim A3* (20 per cent)	0.944	0.956	0.956	1.000	0.998	0.998	0.940
*Sim A3* (40 per cent)	0.944	0.960	0.952	0.998	0.990	0.992	0.958
*Sim B1* (0 per cent)	0.852	0.952	0.960	0.792	0.988	0.988	0.948
*Sim B1* (20 per cent)	0.924	0.956	0.948	0.890	0.974	0.974	0.938
*Sim B1* (40 per cent)	0.930	0.964	0.950	0.940	0.986	0.978	0.942
*Sim B2* (0 per cent)	0.798	0.968	0.960	0.984	1.000	1.000	0.928
*Sim B2* (20 per cent)	0.916	0.960	0.952	0.992	1.000	1.000	0.938
*Sim B2* (40 per cent)	0.940	0.954	0.942	0.986	1.000	1.000	0.938
*Sim B3* (0 per cent)	0.842	0.952	0.954	0.972	0.998	1.000	0.926
*Sim B3* (20 per cent)	0.914	0.958	0.948	0.992	0.996	0.992	0.924
*Sim B3* (40 per cent)	0.946	0.954	0.974	0.996	0.990	0.998	0.924
